# Stabilizing supply of artemisinin and artemisinin-based combination therapy in an era of wide-spread scale-up

**DOI:** 10.1186/1475-2875-11-399

**Published:** 2012-12-02

**Authors:** Rima Shretta, Prashant Yadav

**Affiliations:** 1 Systems for Improved Access to Pharmaceuticals Services, Management Sciences for Health, 4301 North Fairfax Drive, Suite 400, Arlington, VA, 22203, USA; 2 William Davidson Institute, Ross School of Business and School of Public Health, University of Michigan, 724 East University Avenue, Ann Arbor, MI, 48109, USA

**Keywords:** Artemisinin, Artemisinin-based combination therapy (ACT), Safety stock, Buffer stock, Physical buffers, Financial buffers

## Abstract

The global demand for artemisinin-based combination therapy (ACT) has grown sharply since its recommendation by the World Health Organization in 2002. However, a combination of financing and programmatic uncertainties, limited suppliers of finished products, information opacity across the different tiers in the supply chain, and widespread fluctuations in raw material prices have together contributed to a market fraught with demand and supply uncertainties and price volatility. Various short-term solutions have been deployed to alleviate supply shortages caused by these challenges; however, new mechanisms are required to build resilience into the supply chain. This review concludes that a mix of strategies is required to stabilize the artemisinin and ACT market. First, better and more effective pooling of demand and supply risks and better contracting to allow risk sharing among the stakeholders are needed. Physical and financial buffer stocks will enable better matching of demand and supply in the short and medium term. Secondly, physical buffers will allow stable supplies when there are procurement and supply management challenges while financial buffer funds will address issues around funding disruptions. Finally, in the medium to long term, significant investments in country level system strengthening will be required to minimize national level demand uncertainties. In addition a voluntary standard for extractors to ensure appropriate purchasing and sales practices as well as minimum quality and ethical standards could help stabilize the artemisinin market in the long term.

## Background

Since artemisinin-based combination therapy (ACT) was recommended as the first-line treatment of uncomplicated malaria in April 2002 by the World Health Organization (WHO), demand for it has grown sharply [1]. In 2005, WHO [2,3] indicated that demand increased from two million treatment courses in 2003 to 30 million in 2004, to an estimated 70 million in 2005, and a projected 287 million in 2011. By 2011, 84 countries and territories, including all countries in Africa, had adopted ACT as their first-line treatment of *Plasmodium falciparum* malaria [4]. Notwithstanding these policy changes, the rates at which endemic countries have scaled-up the use of ACT have varied, resulting in demand growth which has been difficult to predict accurately.

The global demand for ACT has remained unstable mainly because of financing and programmatic uncertainties. On the other hand, long lead times, a market structure with too few manufacturers at some stages of the production cycle and too many at others as well as information opacity across the different tiers in the supply chain, have led to poor matching of demand with supply. This increasing demand variability moving up the supply chain has created a “bullwhip effect” [5] and the resulting demand and supply imbalances have resulted in widespread fluctuations in raw material prices and in some cases global ACT shortages. In an ideal environment, good country-level data would enable an accurate global-level forecast; however, lacking this data, global forecasts are often based on an intention to procure or projections of donor funds available rather than actual purchases. New initiatives such as the UNITAID-funded ACT forecasting consortium have achieved higher levels of global forecast accuracy than before [6]. However, the current limitations on available information about future funding for ACT impede their ability to improve the accuracy of forecasts far enough in advance to benefit those stakeholders in the supply chain that need them. In years when poor forecasts have contributed to ACT shortage this further reduced the ability to procure the planned quantities. Similarly, in years when there was an over-forecast, it led to over production and decreased prices for raw material resulting in lower overall production in the following years. This cycle of instability has been a hallmark of the ACT supply chain since its adoption.

**Figure 1:**
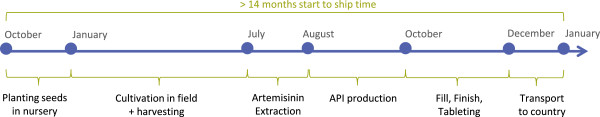
Timeline from planting to availability of final product.

The number of ACT treatment courses procured by the public sector has increased significantly from 11 million in 2005 to 181 million in 2010. ACT demand in 2011 was estimated to be close to 287 million treatments, an increase of 32%over the previous year [4]. Demand has also risen with the introduction of subsidized ACT in the private sector by the Affordable Medicines Facility for malaria (AMFm) [7]. While global demand has grown, the raw material supply market has remained unstable, and there have been reports of significantly lengthened lead times for the delivery of ACT in some years as well as national level stock-outs [7,8]. Short-term solutions have been deployed, such as diversion of shipments to high-risk countries by the United States President’s Malaria Initiative (PMI) and WHO’s ACT supply task force, but new mechanisms are required to ensure better demand and supply matching and to build resilience into the supply chain. Several initiatives have begun to discuss the use of potential “safety nets” to ensure an uninterrupted supply of prequalified ACT [9,10]. However, a detailed and rigorous analysis of the available and feasible options is lacking. This paper attempts to fill that gap.

### Challenges and the history of the global ACT supply chain

*Artemisia annua*, the plant source for artemisinin, is cultivated mainly in China, which with Vietnam, produces approximately 80%of the global supply; East Africa producing nearly all of the remaining 20% [9,10]. It takes about eight months for artemisia to reach full growth and after harvest, dried leaves are sent to artemisinin-extraction facilities [11]. Typically, an extractor contracts with a large number of small farmers for its supply. Specialized active pharmaceutical ingredient (API) manufacturers, or in some cases, the finished product manufacturers convert artemisinin into its derivatives—artemether, artesunate, or dihydroartemisinin—and manufacture the finished product. Figure 1 illustrates the timeline from planting to availability of final product.

The fragmentation of the extraction and API manufacturing market reduces current operating efficiency, which leads to higher transaction costs and amplifies demand uncertainty [12]. In addition, signals to growers and particularly extractors on potential shortages or scarcity of raw material could lead to considerable market disruptions including artemisinin price increases and gouging, as well as product hoarding. This could lead to ACT supply constraints, country level stock-outs and, ultimately, increased finished product prices.

Currently, there are only seven WHO prequalified manufacturers of finished ACT [13]. Global ACT supply issues are caused by a multitude of factors such as limited availability of artemisinin (starting material) and active pharmaceutical ingredient (API), uncertain and fragmented demand for the products, leading to poor forecasts financing delays or shortfalls, and inadequate manufacturing capacity. Uncertain demand results in manufacturers not using their full manufacturing capacity to produce ACT throughout the year. Although manufacturing capacity may seem adequate on an annual basis, an unstable ordering pattern means that manufacturers may allocate production capacity to other products making them unable to fulfill their total annual manufacturing capacity [R. Cazetien, personal communication].

ACT was considered as a viable option for first-line treatment of uncomplicated malaria when a 2001 agreement between WHO and Novartis sought to make Coartem® (artemether-lumefantrine) available at cost to ministries of health in developing nations. This agreement allowed WHO to generate global demand forecasts while providing Novartis with a four-month lead time on all orders [14-16]. Initially these forecasts were too low and in November 2004, WHO announced a shortfall in the supply of Coartem® due to a lack of raw material [16]. Increased production in China and Africa eased this shortage, but then resulted in an oversupply in 2007 and a significant decrease in prices [16]. As a result, many growers of *Artemisia annua* switched to crops with a more dependable demand level.

In July 2008, the Clinton Foundation entered into an agreement with several Chinese and Indian manufacturers that would set price ceilings for artemisinin extractors and help stabilize ACT prices [17]. Separately, a new initiative called the Assured Artemisinin Supply System (A2S2) was created in 2009 to increase artemisinin supply to meet the projected ACT demand in 2010–2012 [8,9]. A2S2 was based on a tripartite financing model where extractors who had existing contracts with WHO-prequalified ACT manufacturers received loan- based pre-financing. The idea was that front-loading the financing would help increase supply and create “fair prices” on the market. However, neither of the interventions has successfully stabilized prices [8,18].

Recent concerns about longer supply lead times on ACT orders led WHO and Roll Back Malaria (RBM) to convene a meeting in September 2011 where participants concluded that the ACT supply was tight, artemisinin prices were soaring, and that there was a need to closely monitor stock levels particularly in countries at risk of shortage. As a result, WHO created the multi-partner ACT Supply Task Force which has been working to ensure that in times of tight supply, country level stock outs can be prevented through better planning and stock reallocation [7].

### Challenges, with the ACT supply chain in malaria-endemic countries

In-country stock-outs result from challenges in procurement, distribution, disbursement delays or supply shortfalls. Currently, countries deal with stock-outs in a largely ad hoc manner. Typically, when a country identifies or anticipates a stock-out, it requests a donor or partner organization to provide ACT stock or short-term financing to procure it. Donors and partners respond in a number of ways to prevent treatment disruptions: countries often transfer stock between districts or facilities to avoid stock-outs or prevent product expiry. Transfer between countries has been implemented among a number of Latin American countries [19] however, this is less common elsewhere. Mechanisms such as the Coordinated Procurement Planning Initiative comprised of the Global Fund to Fight AIDS, Tuberculosis and Malaria (Global Fund) and a number of partners have been established to develop a joint and collaborative response to potential and actual stock-outs for HIV commodities [20].

Poor matching of demand and supply, financial flow uncertainty, and poor country level estimates of demand also contribute to the cycle of instability. Although discussed separately below, the three are interlinked, and problems in one contribute and lead to problems in the others.

### Financial delays

A large number of countries rely on Global Fund financing for procuring ACT [21]. Public sector procurement using Global Fund grants contributes to more than 1/3 of the global ACT market [22]. A major financial reason for unpredictable ACT country demand is funding gaps caused by delays in disbursement by donors such as the Global Fund. Lengthy cycles for grant review and approval, delayed responses from the Principal Recipient on disbursement requests and progress updates, and linking disbursements for commodity procurement to poor grant performance contribute to delayed grant disbursement and tardy procurement [23,25].

An assessment of Global Fund grants by the African Leaders Malaria Alliance (ALMA) illustrated that funding delays consistently led to procurement delays of malaria commodities in at least nine countries in 2011 [M. Renshaw, personal communication]. Key delays have been related to grant signature, grant consolidation, and transitioning to phase 2 of grants. Other reports have noted delays with quarterly disbursements due to issues with the reporting of performance indicators [26]. For example, in Uganda, no disbursements were made from May 2010 to June 2011 leading to widespread ACT stock-outs. In Mali, PMI averted a stock-out caused by delays in grant consolidation and signature leading to disbursement delays by placing an emergency ACT order. An analysis of PMI procurements carried out by the USAID | DELIVER PROJECT between October 2007 and October 2011 revealed that PMI funded the procurement of 54.3 million ACT treatments to fill Global Fund and other donor funding gaps [L. Hare, personal communication].

Many countries rely on resources being disbursed within six months after grant approval, and according to the Global Fund’s policies, the signature process cannot extend beyond one year. However, on average, it takes at least 340 days [28] from the time of grant approval to grant signature, resulting in procurement and programmatic delays. For example, in Ghana, the delay in receipt of funds from both the Round 5 Rolling Continuation Channel and Round 8 caused delays of up to 12 months in indoor residual spraying (IRS) and long-lasting insecticide treated net (LLIN) prevention programmes as well as in the rollout of community case management of malaria [27]. The Global Fund Board at its meeting in November 2012 recommended that the AMFm will also be incorporated into the existing grant mechanism, which may add to the uncertainties of funding flows for procurement of ACT [28].

### In-country procurement and supply chain management challenges

Inefficient procurement and supply chain management processes in countries lead to challenges with reporting and meeting targets, which also add to Global Fund grant disbursement delays. A study carried out by the Roll Back Malaria Procurement and Supply Management Working Group estimated that the average time taken to complete the steps in procurement until a purchase order was placed was six months for malaria commodities [29]. Furthermore, the procurement process can be complicated by corruption in some countries; in 2011, 12 grants were investigated by the Global Fund Office of the Inspector General for poor procurement practices [30].

Despite recent commitments by partners and countries to strengthen the supply chains and to improve ACT forecasting, challenges remain because of inherent weaknesses, in the health system including poor stock management and warehousing; inconsistent, inaccurate, or unavailable data for forecasting and decision-making; and weak human resource capacity and program coordination [31]. The absence of functioning management information systems also impedes the early detection of stock-outs. Idiosyncrasies of the product itself, including short shelf life and a requirement to order ACT packs according to patient weight, are additional complications.

### Fluctuating demand

The epidemiology of malaria is changing rapidly because of effective malaria control interventions. Several reviews [32] have demonstrated that ACT scale-up and vector control measures such as IRS and LLINs have reduced parasite transmission and malaria cases in many countries. Furthermore, many countries are scaling-up diagnostic testing programmes for malaria with microscopy or rapid diagnostic tests. However, the proportions of cases that undergo diagnostic testing and are subsequently treated with ACT are not well documented. The quantifiable short- and medium-term effect of these interventions on the demand for ACT is, therefore, unclear [33].

**Figure 2:**
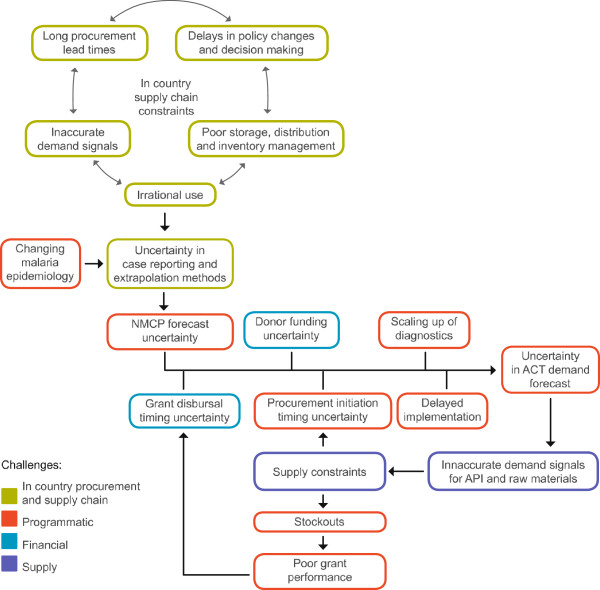
Vicious cycle of demand and supply uncertainty in the ACT supply chain perpetuated by supply chain challenges at the country level.

Natural disasters including changing rainfall patterns, floods, droughts, and epidemics also give rise to uncertainties. For example, Kenya has recently experienced the worst drought in 60 years, which resulted in malaria outbreaks in the arid north and highlands when the rains returned. This sudden increase in demand led to a delayed delivery of ACT and stock-outs at all levels as manufacturers had not planned for these sudden orders [7]. Figure 2 illustrates the vicious cycle of demand and supply uncertainty in the ACT supply chain perpetuated by supply chain challenges at the country level.

### Strategies to cope with demand and supply uncertainty in the ACT market

While several of the nuances in the ACT supply chain are unique, the situation of matching supply with demand in environments with high uncertainty translates to many other products and geographical contexts. Approaches that can be used to address the demand and supply uncertainty in the ACT supply chain are presented below along with a discussion of the pros and cons of each approach. In addition to short and medium term options, some long term options are also analysed that could help stabilize the artemisinin market. The options are illustrated in Additional file 1.

### Effective pooling of demand and supply risks

The volatility in ACT demand is greater at the individual country level as compared to a region with multiple countries. A supply chain can better manage demand volatility by aggregating demand across multiple customers or geographical areas thereby smoothing short-term ordering fluctuations. Similarly, the impact of supply uncertainty is greater when there is a single supplier. During instances of oscillating demand, pooling products from multiple suppliers can lead to better matching. Using careful planning to match demand and supply has proven beneficial in several pooled procurement initiatives [34-36]. Although the Global Fund Voluntary Pooled Procurement mechanism was created to achieve such benefits, it has not been able to achieve its full potential because of partial implementation. The Global Fund now intends to “fully” implement VPP including opt-out instead of opt-in mechanisms [37].

### Rolling horizon forecast /volume commitments

A rolling horizon forecast commitment requires funding agencies such as the Global Fund to commit to manufacturers to purchase a certain quantity of the product over two-three years with some flexibility in updating the commitment as new information becomes available. In return for the flexible purchase commitment, the manufacturers would guarantee a maximum allowed lead-time, upside purchase flexibility, and lower prices. Explicit contractual penalties are defined for the manufacturer if it cannot meet either its lead-time commitment or the upside supply guarantee. Thus, the funding agency reduces its risk of supply shortage and price uncertainty by undertaking some of the demand uncertainty risk [38-40]. Volume commitments from funding agencies to manufacturers would result in more equitable sharing of the risk from demand uncertainty across the different stakeholders in the supply chain (e.g., ACT manufacturers, funders, country governments, extractors and API manufacturers) [39,40]. In some cases volume commitments would enable a switch in the manufacturer’s production paradigm from “make to order” to “make to stock” resulting in reduced lead times.

The level of flexibility in the commitments at each stage and any contractual penalties need to be chosen carefully based on a thorough analysis of the forecast certainty and risk aversion ability. The manufacturer would continue to experience risk associated with exact timing of order placement within the year, although the concept of a demand-driven supply network described in the following sections attempts to minimize some of this risk. A rigorous mathematical analysis of rolling horizon forecast commitments used for non-health commodities can be found in Anupindi and Bassok [41]. The United Nations Children’s Fund (UNICEF) uses similar contracting arrangements called long-term arrangements where solid forecasts are established between manufacturers and UNICEF with some flexibility in changing them [42-45].

In some cases the funding agencies may themselves not have a predictable multi-year financing stream in order for them to make future volume commitments to manufacturers. Additionally, the nature of the ACT market is very dynamic; new ACT formulations and new types of anti-malarials as well as new manufacturers enter the market space every few years and prices have fluctuated significantly. This has made some buyers reluctant to make multi-year purchase commitments due to the perception that this may leave them with fewer degrees of freedom.

### Global supply hubs/physical buffer stock

Holding some ACT inventory in a central location to fill orders that have a short lead-time allows some decoupling between the fluctuations in ACT demand and the uncertainties in ACT supply. Buffer stocks can be held by manufacturers or third parties and can be either rotating or non-rotating. Rotating product buffers provide the benefits of short lead times, risk pooling, discounted pricing, and a low risk of product expiry, but can be challenging to implement. Non-rotating emergency buffers are easy to establish and enable rapid response; however, they also present a high risk of expiry if the amount of stock is not estimated properly.

Typically, manufacturer-held buffers have the lowest cost and best shelf life and are particularly effective with products with cold chain requirements. Holding buffers at an upstream level in the supply chain leads to lower total inventory costs due to statistical aggregation of uncertainty as well as lower costs of warehousing and inventory holding at the manufacturer warehouse. However, manufacturers are often reluctant to assume liability for stock, especially without guaranteed funding or the existence of a secondary market if demand is lower than expected. To ensure an effective manufacturer-held buffer, funding must be secure or guaranteed. Also, in instances where a mix of products is required, holding buffer stock across multiple manufacturers introduces an additional layer of complexity requiring countries to receive multiple shipments for each order which increases transit costs and the risk of product loss and delays.

With a third-party buffer stock, the initial stockpile is financed using a rolling working capital fund that is replenished when orders are placed. The supply hub would carry inventory from all WHO pre-qualified manufacturers in proportion to their market share in the last year and would be located in a region that provides easy logistical access to malaria-endemic countries. The manufacturers would then replenish the stocks at the supply hub as orders deplete it. This implies that a higher fraction of the supply chain will be demand driven and not based on a forecast alone, and manufacturers would not wait for production activities to start after the order is received. To avoid any contractual penalties stipulated in the rolling horizon forecast commitment, the manufacturers will continue to hold inventory. On the other hand, because of short shelf life, holding excess inventory will be expensive for the manufacturer. These two forces together will incentivize the manufacturers to hold the optimal quantity of stock creating the balance required to match supply with demand in the short-term. A regionally located inventory can also result in lower transport costs and lead to better preparedness for any time-sensitive, short-term demand spikes that may require more than one supply source, such as a malaria epidemic. Such stockpiles and buffers have been successfully used by PEPFAR/SCMS [46-48] for HIV medicines, PMI for ACT [49], the Global Drug Facility (GDF) for Multi Drug Resistant Tuberculosis (MDR-TB) medicines [50] and UNICEF for multiple vaccines [43-45].

Physical buffers are not difficult to manage and provide the ability for short lead times while allowing price advantages from pooled procurement; however, countries can become dependent on the stockpile and place all orders as emergencies. There is anecdotal evidence from some of the above initiatives that normal procurement constitutes only a small percent of all procurement, while the remainder goes to replenish the stockpiles for emergency orders. Furthermore, although manufacturers are more likely to agree to hold buffer stocks when they share the risk of product surpluses additional mechanisms to ensure timely delivery of products should be established.

Because of the long production cycle for ACT, a fundamental question is which stage of the supply chain the buffer inventory should be held—artemisinin harvest, API, or finished product? The answer depends on who pays for the buffer inventory and the existence of product quality standards for analysing finished product quality. Manufacturers routinely hold their own buffer inventories of raw materials, API, and to some extent, finished product. If they have adequate incentives, for example, through forecast commitments, manufacturers could hold higher amounts of inventory at each of those stages. The ACT manufacturing process is however, market driven with heterogeneity in the quality of raw material used (within certain tolerance limits specified under regulatory filing), and each manufacturer may have process parameters that have been stabilized over a period of time to work with varying impurity levels. Therefore, a centrally held API or raw material buffer is not feasible even though it may be theoretically more desirable. If a publicly funded buffer inventory exists to meet short-term mismatches in demand and supply at the country level, the buffer inventory should comprise finished products and not raw material or API.

### Financial buffer fund for ACT

Financing buffers including emergency contingency funds can be very effectively used to initiate ACT procurement on time when temporary shortfalls in funding threaten supply disruption. For example, the PEPFAR and PMI programmes have established emergency commodity funds that provide countries with rapid access to financial resources in the event that Global Fund disbursements are delayed [47,49]. Countries accessing the buffer are expected to replenish the fund as resources become available. UNICEF also uses a similar line of credit for vaccines [45].

Another example of an initiative that provides bridge financing to grant recipients on the basis of pending aid commitments so that they can procure essential commodities while awaiting donor funding is the Pledge Guarantee for Health, conceived by the Bill & Melinda Gates Foundation, the Reproductive Health Supplies Coalition, Dalberg Global Development Advisors, and the United Nations Foundation [51].

Financial buffers are easy to implement and may require less oversight and administrative burden compared to product buffers. Countries accessing financing buffers have the freedom to select the manufacturer and product that best meet their requirements and are not limited to just stock-piled products. Challenges associated with establishing financing buffers include a significant risk of de-capitalizing the buffer due to repayment default by a country. Decisions to release funds must be made quickly, and as a result, sufficient due diligence may not always be possible. Financial buffers rely on the finished product being readily available and do not present any of the lead time or pricing benefits that can result from establishing a physical buffer. In addition, they create a parallel mechanism for releasing funds; bypassing and potentially undermining existing processes as well as removing the mechanism to enforce conditions related to under-performance. Creation of a separate mechanism rather than tapping into an existing structure also requires seed financing and the identification of a body to host and implement the mechanism.

### Options for stabilizing the artemisinin market in the long term

In addition to better matching of demand and supply for ACT, it is imperative to stabilize the market for artemisinin. Several methods for doing this can be considered but each requires a medium- to long-term impact outlook and may not be implementable in the short-term.

Sanofi-Aventis is expected to launch a semi-synthetic variant of artemisinin in 2013 [52] which would reduce the lead time of artemisinin from the current 7 months agriculturally to only a few days in the reactor. While exact figures on price and quantity available are not reported in any published literature, according to recent reports [12] an estimated 50 metric tons of semi-synthetic artemisinin will be available in 2013 at a price between US$ 350-400/kg. While this technology has the potential for stabilizing the market if scaled-up, the supply in the immediate term will only have a small impact on the overall artemisinin market. Therefore, other complementary interventions will still be required to address the uncertainties in the artemisinin market.

### Voluntary standards for extractors

To bring greater transparency to the artemisinin extraction and the leaf collection business, a voluntary standard could be created for extractors that would involve adherence to both appropriate purchasing and sales practices and minimum quality or impurity thresholds. Such a standard could be established by an independent standard-setting agency or a consortium of partners that would monitor the extractors to ensure socially responsible business practices and quality in the artemisinin production process.

Large purchasers such as PMI and Global Fund would then ensure that ACT manufacturers from whom they purchase source their artemisinin only from extractors who have opted to meet the voluntary quality standard. This standard would reduce the number of participating extractors and in turn simplify the API supply chain and result in fewer, and more responsible, extractors that meet standards of product quality as well as better selling and pricing practices [12]. This mechanism would not be intended to standardize artemisinin quality specifications across all manufacturers, but instead would only allow extractors who engage in responsible sourcing, pricing and selling practices. This would create both price stability in the market as well as allowing manufacturers to engage in forward purchasing without the risk of extractors reneging their contracts.

### Better information sharing mechanism across the supply chain

In the long term, if agriculturally grown artemisia is to be the main source of artemisinin, a robust mechanism needs to be developed that leads to better information sharing among farmers, extractors, and manufacturers regarding artemisia growth, artemisinin sales, and forecasts. Decision-making across the ACT supply chain needs to become less reliant on the intermediaries and market signals received from single parties. A technology-supported information exchange platform should be designed that can provide anticipated demand information directly to artemisia farmers in the main growing areas. Farmers in artemisia growing areas can receive up-to-date information on demand, climate change, and farming practices, and communicate their needs to other invested parties [12]. Such methods of providing better demand and price information to farmers have been commonly used to eliminate middle-men and stabilize prices in other agricultural sourcing markets including the ITC e-Choupal programme [53].

## Conclusions

Universal access and use of diagnostics would help us understand the true burden of malaria and allow better visibility of the true demand for ACT creating a better forecast. Accurate forecasting information from the country level will enable a closer matching of global demand and supply; however, this will require significant investments in national health system strengthening which are unlikely in the short-term. Better grant management and disbursement at the Global Fund as a part of its new funding model will also relieve some of the pressures on the ACT supply chain, especially with respect to financing delays. However, many challenges will persist and a mix of strategies will be required to stabilize the artemisinin and ACT market.

Physical buffer stocks are best suited to deal with procurement and supply management and global supply challenges, while a financial approach better addresses funding disruptions. For example, if a stock-out is created because a country does not have the funding available to purchase products when needed, access to readily available financial resources would be more beneficial than access to a physical buffer (which the country may not be able to draw from in the absence of confirmed or guaranteed capital).

On the other hand, poor procurement and supply management capabilities may prevent a country from anticipating stock-outs far enough in advance for a financial option to be effective. In this case, a physical product buffer would be more appropriate, since product would be readily available and can be quickly accessed.

While the availability of synthetic and semi-synthetic artemisinin may ease some market pressures [52], the projected quantity to be produced in the short-term [12] is unlikely to create a significant rebalancing of the supply chain. Therefore, a physical regional buffer held by a third party to fill orders with short lead-time windows will be needed to provide a temporary solution. Seed financing will be needed to develop the initial stockpile, which can be replenished when orders are placed, enabling a supply chain linked to both forecasts and actual demand. Efforts must be made to create a rolling horizon forecast commitment model that is feasible within the legal boundaries of the donors. Such a model would incorporate minimum but flexible purchase quantities over a defined period of time that embodies the core principles of the strategies outlined. The regional buffer should be considered short to medium term solutions. Developing better medium- to long-term investments to streamline country-level processes and supply chain management would ultimately make such a buffer redundant. In addition, for countries that are faced with significant disbursement delays, financial buffers that provide bridge funding need to be considered. These financial buffers must ensure a short lead time to mobilise resources for procurement.

In the medium to long term, to bring greater transparency to the artemisinin extraction and the leaf collection business, a voluntary standard should be created for extractors to ensure appropriate purchasing and sales practices. In addition, minimum thresholds should be established leading to high quality products and an adherence to a minimum ethical standards. At the same time, significant investments in country level system strengthening will be required to minimize demand uncertainties at the national level. Streamlining donor processes for fund disbursement and developing better information exchange standards among farmers, extractors, and manufacturers from the agricultural process to sales and purchasing will prevent the bullwhip effect arising from country level demand variability to ensure that decision making is not based on inaccurate market signals and ultimately enable a better matching of supply and demand.

## Abbreviations

ACT: Artemisinin-based combination therapy; ALMA: African Leaders Malaria Alliance; AMC: Advanced market commitment; AMFm: Affordable Medicines Facility for malaria; API: Active pharmaceutical ingredient; ARV: Antiretroviral; GDF: Global [TB] Drug Facility; Global Fund: Global Fund to Fight AIDS, Tuberculosis and Malaria; ICG: International Coordinating Group; IRS: Indoor Residual Spraying; LLIN: Long Lasting Insecticide Treated Nets; PEPFAR: US President’s Emergency Plan for AIDS Relief; PMI: US President’s Malaria Initiative; RBM: Roll Back Malaria; SIAPS: Systems for Improved Access to Pharmaceuticals and Services Program; UNICEF: United Nations Children’s Fund; USAID: US Agency for International Development; WDI: William Davidson Institute; WHO: World Health Organization.

## Competing interests

The authors declare that they have no competing financial or non-financial interests.

## Authors’ contributions

RS and PY drafted the outline and the manuscript and participated in its design. RS coordinated inputs to the draft. All authors have read and approved the final manuscript.

## Supplementary Material

Additional file 1 Analysis of approaches for better matching of ACT demand and supply. Click here for file
