# Supplementary material for: Stabilizing supply of artemisinin and artemisinin-based combination therapy in an era of wide-spread scale-up
Source: Malar J. 2012 Dec 2;11:399. doi: 10.1186/1475-2875-11-399 (PMC3556147; doi:10.1186/1475-2875-11-399)
Supplement: Additional file 1 — Analysis of approaches for better matching of ACT demand and supply. [file 1475-2875-11-399-S1.docx]

**Table 1: Analysis of Approaches for Better Matching of ACT demand and supply**

|  | Rolling Horizon Forecast Commitments | Pooling demand and supply risks | Physical Buffer Stocks | Financial Buffer Funds |
| --- | --- | --- | --- | --- |
| Basic Premise | Funding agencies commit to manufacturers to purchase a certain quantity of ACT over a fixed time frame with some flexibility. Manufacturers guarantee a maximum lead-time, upside purchase flexibility, and lower prices | Volatility in ACT demand is lower when demand is aggregated. Pooling supply from multiple suppliers can reduce the supply side uncertainty | Holding some inventory in a central location (usually closer to demand) to fill orders that have a short lead-time | Holding funds in a buffer account to bridge disbursement delays that can cause serious stock outs |
| Design Questions | - Duration of commitment (2 year, 3 year, 5 year)? - Flexible vs. rigid commitment | - Regional or global pools? - Voluntary or mandated pools? | - Level in the supply chain to hold buffer: artemisinin, API, or finished product - Rotating or non-rotating buffer - Held by manufacturer or funding agencies - Initial distribution of stock in the buffer from different manufacturers | - Conditions or risk profile for disbursing from financial buffer |
| Recommended Option | - 3 year commitment with no quantity flexibility for Year 1, 20% flexibility for Year 2 and 50% flexibility for Year 3 | - Voluntary global pools | - Finished product ACT buffer - Held by funding agencies - Rotating buffer - Last period market share based initial allocation in the buffer | - Develop stock out risk metrics to determine eligibility |
| Benefits | - Reduces risk of supply shortage and price uncertainty for the funding agency - Reduces risk of demand uncertainty for the manufacturers. - Lower prices, reduced lead-times, fewer stock outs | - Reduces risk of supply shortage - lower prices, reduced lead-times, fewer stock outs | - Reduced risk of stock outs - Reduced lead-times for orders filled from stockpile. - Reduced transportation costs in some cases | - Reduces risk of stock outs due to late disbursement. - Reduces costs resulting from emergency procurement/shipments |
| Risks/Potential Disadvantages | - The nature of the ACT market is very dynamic; new ACT formulations and new types of anti-malarials enter the market every few years, new manufacturers enter the market and prices fluctuate significantly. Multi-year volume commitments may limit opportunities for funders | - Only one or two purchasers in the ACT market creates a monopsony in the market | - Countries can become dependent on the stockpile and place all orders as emergencies | - Less effort is exerted to ensure that grant management and disbursement are functioning well - Risk of non-payment - Decisions to release funds has to be made quickly, not allowing detailed due diligence |
| Pre-requisites | - Good forecasts | - Working capital to pool purchase orders before each purchaser releases funds |  |  |
| Examples | - UNICEF long- term agreements with manufacturers; Advanced Market Commitment (AMC). | - Global Fund VPP - PAHO regional group purchasing of vaccines. | - PMI; PEPFAR/SCMS UNICEF (vaccines), Global Drug Facility (GDF) 2nd-line TB drugs. | - PEPFAR/PMI emergency commodity fund; PGH; UNICEF line of credit |
